# Temperature Differences Between Rooftop and Urban Canyon Sensors: Diurnal Dynamics, Drivers, and Implications

**DOI:** 10.3390/s25134121

**Published:** 2025-07-02

**Authors:** Lorenzo Marinelli, Andrea Cecilia, Giampietro Casasanta, Alessandro Conidi, Igor Petenko, Stefania Argentini

**Affiliations:** 1 Physics Department, University of Rome Tor Vergata, Via della Ricerca Scientifica 1, 00133 Rome, Italy; lorenzo.marinelli.18@alumni.uniroma2.eu; 2 National Research Council of Italy, Institute of Atmospheric Sciences and Climate (CNR-ISAC), Via Fosso del Cavaliere 100, 00133 Rome, Italy; g.casasanta@isac.cnr.it (G.C.); i.petenko@isac.cnr.it (I.P.); s.argentini@isac.cnr.it (S.A.)

**Keywords:** urban temperature, sensor siting, rooftop measurements, urban canyon, WMO compliancy

## Abstract

Understanding temperature variations within the complex urban canopy layer (UCL) is challenging due to limitations and discrepancies between temperature measurements taken in urban canyons and on rooftops. The key question is how much these measurements differ and what factors contribute to these differences. According to the guidance by the World Meteorological Organization (WMO), rooftop observations are not encouraged for urban monitoring, due to potentially anomalous microclimatic conditions, whereas measurements within urban canyons are recommended. This is particularly relevant given the increasing number of rooftop sensors deployed through citizen science, raising questions about the representativeness of such data. This study aimed to address this knowledge gap by comparing temperatures within the UCL using two sensors: one located on a rooftop, and the other positioned within the canyon. The temperature difference between these two nearby locations followed a clear diurnal cycle, peaking at over 1 °C between 12:00 and 16:00 local time, with the canyon warmer than the rooftop. This daytime warming was primarily driven by solar radiation and, to a lesser extent, by wind speed, but only under clear-sky conditions. During the rest of the day, the temperature difference remained negligible.

## 1. Introduction

The growing use of citizen science and low-cost sensor networks for urban climate monitoring has led to a rapid increase in temperature measurements collected from rooftop-mounted instruments. While these locations are often the most accessible and practical for deployment—especially in densely built environments—they raise important questions about the representativeness and reliability of the data collected. In particular, the guidance by the World Meteorological Organization (WMO) [1] explicitly discourages the use of rooftop sites for standard meteorological observations in urban environments. According to this reference, rooftops are characterized by anomalous microclimatic conditions—including strong vertical thermal gradients, irregular wind fields, and atypical surface properties—that may compromise the reliability of temperature and humidity measurements. According to this guidance, rooftop measurements are considered compliant with standard observational criteria only under strict conditions. Specifically, it recommends that, when rooftop installation is unavoidable, sensors should be placed at a height of at least 1.5 times the average height of the surrounding buildings, to ensure that the measurements adequately reflect the mixing between roof-level and canyon-level air. However, even for buildings with an average height of 10 m, this would imply mounting a 5-meter mast on the rooftop, an operation that is virtually unfeasible in practice, especially for private citizens or in citizen-led monitoring initiatives.

Instead, the WMO recommends placing instruments within urban canyons. For non-urban environments, sensors must be installed between 1.25 m and 2 m above ground level. In urban settings, however, the upper limit is relaxed, even allowing for placements up to 5 m, given the minimal vertical temperature gradients typically observed within street canyons.

However, recent studies have begun to challenge this perspective [2,3]. In particular, Cecilia et al. [4] demonstrated that rooftop-based measurements in Rome, even when taken just 2 m above the roof surface, could yield consistent and spatially representative temperature data, with an estimated footprint on the order of 1 km. These data were also successfully used to quantify the intensity of the urban heat island (UHI), producing results that are fully consistent with previous studies based on WMO-compliant instrumentation, thereby supporting their applicability in urban climate research.

Notably, both rooftop measurements at 2 m height and those taken within urban canyons fall within the vertical extent of the urban canopy layer (UCL). The UCL refers to the atmospheric layer extending from the ground up to the average building height, encompassing streets, open spaces, and elements such as trees or vegetation located within this volume [5,6]. It plays a key role in shaping local microclimatic conditions. By analyzing temperature variations, valuable insights can be gained into how different components of the urban canopy affect the thermal environment. Detailed studies and precise measurements of the UCL’s thermal dynamics enable the implementation of targeted solutions to optimize the well-being of urban residents and improve the overall quality of life in metropolitan areas [7,8].

Temperature measurements within urban street canyons are strongly influenced by local morphological characteristics, including canyon geometry, street orientation, building density, sky view factor (SVF), surrounding materials, vegetation, and wind dynamics [9,10,11]. These factors can vary substantially from one canyon to another, leading to pronounced spatial heterogeneity in temperature and radiative fluxes [9,10,12]. As a consequence, such measurements typically exhibit a limited spatial footprint and are less suitable for capturing broader urban thermal patterns.

In addition, the radiative energy balance within street canyons differs from that on rooftops. Canyons are subject to reduced sky view factors and multiple radiative reflections between building surfaces, which alter the net radiative fluxes and consequently affect both surface and air temperatures within the canyon volume [13]. Ventilation within canyons is also modulated by the interaction between canyon geometry and prevailing wind direction, often resulting in reduced airflow, lower turbulence, and limited dispersion of heat and pollutants [14]. All these factors make temperature measurements within urban canyons highly localized and difficult to compare across different sites.

Conversely, rooftop stations are typically located at the top of the UCL, where they receive more direct solar radiation and are more exposed to wind. As a result, they are generally less influenced by the immediate urban morphology compared to canyon-based sensors, making them more spatially representative and suitable for broader-scale assessments [4]. Furthermore, rooftops are often the most accessible and practical locations for sensor deployment, especially in the context of citizen science and large-scale urban monitoring networks. Given the growing number of these rooftop stations worldwide, aligning their installation criteria with WMO observational standards would be highly beneficial, potentially unlocking a valuable and currently underutilized source of urban climate data.

Some studies have investigated temperature differences between rooftop and canyon measurements. Field experiments in Trento [15], Italy, confirmed that the temperature variations within an urban canyon were primarily driven by solar radiation and surface heating, with notable differences depending on wall orientation and time of day. For instance, during nighttime or overcast conditions, the temperature distribution became more homogeneous. Similar results, with the canyon being consistently warmer than the rooftop, were also obtained in Gothenburg, Sweden by Offerle et al. [16]. However, in vegetated canyons—where shading reduces surface exposure to solar radiation—surface temperatures tend to be lower than those on rooftops, especially during daytime hours [10,17,18].

Understanding temperature variations within the UCL is crucial for analyzing urban climate dynamics, particularly the urban heat island (UHI) effect. This phenomenon, typical of urban environments, refers to the higher temperatures observed in cities compared to their rural surroundings, due to human activities and the characteristics of the built environment. This warming is caused by heat retention in urban elements such as asphalt, buildings, and other impervious surfaces, as well as reduced vegetation and changes in airflow [19,20,21]. Within cities, temperature variations are strongly influenced by urban geometry, with street canyons trapping heat due to limited ventilation and multiple reflections of radiation between buildings [22].

The intensity of the UHI effect can vary significantly depending on the measurement location, as the local urban structure greatly influences the recorded values [10]. Ensuring that measurements are representative at the neighborhood scale allows for a more accurate interpretation of the collected data.

Building on these considerations, the present study aimed to evaluate to what extent the official siting criteria can be relaxed by directly comparing observations from a rooftop station and a co-located sensor within a representative street canyon. Although rooftop and canyon measurements have been investigated separately in previous studies [2,4,16], the direct analysis of their temperature difference over extended periods remains limited. The innovative aspect of this work lies precisely in the systematic quantification of the rooftop–canyon temperature contrast, assessed across diurnal and seasonal cycles, and interpreted in relation to meteorological drivers such as solar radiation and wind. Ultimately, the goal is to assess whether rooftop observations can be considered a valid alternative for UCL monitoring in both operational and research contexts.

In this paper, we characterized the temperature difference between a weather station located on a rooftop and one situated within an urban canyon, analyzing the presence of possible diurnal and seasonal cycles. We also investigated the role of wind speed, wind direction, and solar radiation in shaping this temperature difference, to better understand the drivers of urban microclimatic variability. An approach to estimate the measurement uncertainty associated with hourly averaged temperature values is also presented, as the standard deviation of the original data was not representative of the uncertainty when the variable exhibited an hourly trend.

The broader aim of this study is to support the assessment of the UHI effect by evaluating the reliability of rooftop-based weather stations, such as those from the ASTI network available across the city of Rome, and to determine how the choice of sensor placement may influence the measurement of air temperature in urban areas.

## 2. Materials and Methods

### 2.1. Site and Measurements

The district of Rome, with approximately 2.8 million inhabitants, is the most populous in Italy and among the largest in the European Union. The city spans an area of 1287 km^2^ and features a diverse orography, with altitudes ranging from sea level to over 100 m a.s.l. in the southeastern sectors. Rome has a Mediterranean climate (Csa in the Köppen classification) [4,23], characterized by mild winters and hot, dry summers. Local wind regimes are dominated by daytime sea breezes from the Tyrrhenian Sea, which mitigate urban temperatures, especially in the western districts. At night, the local circulation is more complex due to the city’s morphology, which includes a mix of large green areas and densely urbanized zones, giving rise to localized nocturnal breezes that vary across different parts of the city. These circulations significantly influence the thermal behavior of the urban atmosphere [4,24,25].

**Figure 1:**
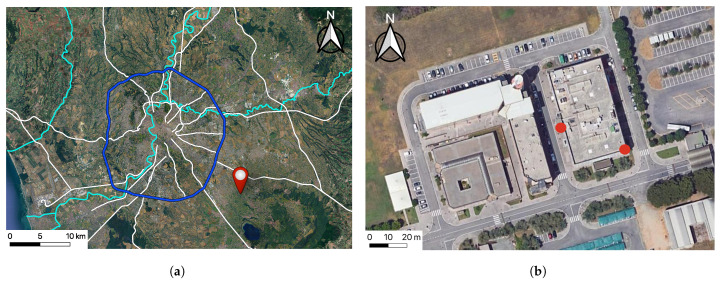
(**a**) Map of Rome and its surroundings highlighting the location of the CNR-ISAC building. The blue line represents the GRA highway encircling the city, white lines indicate major roads, and rivers are shown in light blue. (**b**) Orientation of the street canyon with the North direction indicated. The positions of the two measurement stations are marked with red dots.

The experiment was conducted at the National Research Council of Italy (CNR) Rome Tor Vergata research center (41°50′25.1″ N, 12°38′53.1″ E) from July 2023 to March 2025. Two weather stations were installed on the roof of a building and in an urban canyon, located a few dozen meters apart, in a semi-rural area (20 km from the city center of Rome, Figure 1a) surrounded by buildings of varying height.

**Figure 2:**
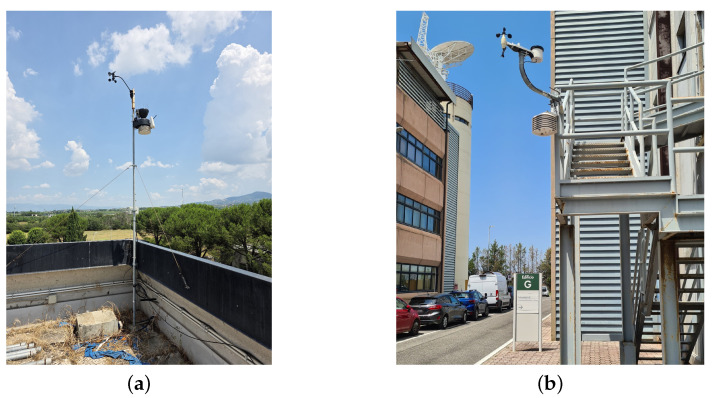
(**a**) Davis Vantage Pro 2 station installed on the rooftop, with a 2.5 m support frame above the surface. (**b**) Froggit WH1080S station installed within the canyon at a height of 3 m above ground level.

The station installed on the roof is a Davis Vantage Pro 2 (Davis Instruments Corp., Hayward, CA, USA) Wireless, equipped with a thermo-hygrometer in a passive radiation shield. It is mounted at the edge of the roof, as shown in Figure 2a, at a height of 2.5 m above the roof surface. This height ensures that the sensor remains fully exposed to direct solar radiation throughout the day. Despite the presence of a slightly taller adjacent building, this mounting height is sufficient to prevent shading during the afternoon hours, as confirmed by on-site inspection. The nominal accuracy associated with the point temperature measurement is 0.3 °C.

The station in the canyon is a Froggit WH1080SE (Froggit GmbH, Bad Salzuflen, Germany) (Figure 2b), which has been modified to achieve measurement accuracy comparable to that of the rooftop station. Although it would have been preferable to use identical instruments, the canyon station had been installed prior to the rooftop deployment and could not be replaced. To ensure comparability, the original sensor was replaced with an SHT35 thermo-hygrometric probe, which has a nominal point temperature measurement accuracy of 0.3 °C. This sensor was also placed inside an 8-plate Davis passive radiation shield, which was positioned 3 m above the street and 2 m away from the adjacent building wall. The street canyon, visible in Figure 1b, has a length of 120 m, an average building height of 15 m, and a width of 13.5 m, resulting in an aspect ratio (λ) of 1.1. The measured orientation of the canyon’s main axis is 345°. Compared to previous studies based on distributed sensor networks or short-term campaigns [2,4,15], the present setup adopted a more controlled and targeted configuration. Although different sensors were used, a dedicated calibration procedure ensured that the temperature difference between rooftop and canyon could be reliably characterized.

### 2.2. Weather Station Calibration Procedure

**Figure 3:**
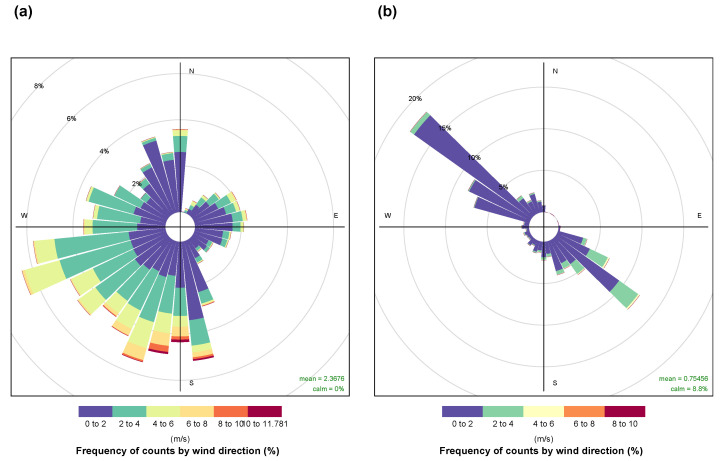
Wind rose diagrams for the stations installed on the rooftop (**a**) and inside the canyon (**b**), showing wind direction and frequency for the two locations.

The wind roses shown in Figure 3 characterize the measurement site in terms of wind direction, speed, and frequency for both the rooftop station (a) and the canyon station (b). The wind roses are based on hourly measurements collected from July 2023 to March 2025, covering the full duration of the monitoring campaign.

In summer, during the central hours of the day (12:00–17:00), the prevailing wind regime is dominated by sea breeze, with winds from the W–SW direction, typically ranging between 3 and 5 m s^−1^. Another frequently observed wind direction is from the North, with wind speeds typically ranging between 1 and 3 m s^−1^. At night, a land breeze develops predominantly from the South-Southeast (S–SE), with wind speeds between 1 and 4 m s^−1^.

The wind rose for the station located within the canyon (Figure 3b) clearly shows its orientation along the 345°–165° axis, as determined from on-site geometric measurements. Additionally, it also highlights that the wind speeds measured at the rooftop were consistently higher than those observed within the canyon. This difference is mainly due to the influence of edge effects and lower aerodynamic obstruction at rooftop level compared to the constrained geometry of the canyon [14].

### 2.3. Weather Station Calibration Procedure

Both the Froggit WH1080SE and the Davis Vantage Pro 2 stations were calibrated using a CH600C climate chamber by Angelantoni Industrie Srl. (Massa Martana, Italy). The temperature inside the chamber varied from −25 °C to 50 °C with 5 °C step. To account for potential differences in sensor equilibration times, only temperature values corresponding to stable plateau were retained. Data points were excluded when the difference between consecutive temperature readings exceeded 0.2 °C, indicating a transient phase.

This filtering process was necessary to remove temperature transients within the climate chamber and to compare the two temperature series once a stable plateau was reached. A regression analysis was then performed to intercalibrate the datasets, taking the Davis station as the reference. The resulting calibration equation was applied to the temperature series measured by the Froggit station to correct its tendency to underestimate temperature values.

The calibration followed a linear model of the form

(1) $$T_{\mathrm{Davis}}=\alpha \;T_{\mathrm{Froggit}}+B,$$

where α=1.0 and B=0.65 °C. This correction was applied to the entire Froggit temperature time series.

### 2.4. Uncertainty Evaluation

Hourly averages were determined for both the temperature series, and the temperature differences between the measurements recorded by the Davis station on the rooftop and the Froggit WH1080SE station within the canyon were subsequently calculated.

As noted by Pérez Ballesta [26], characterizing uncertainty in a time series of measurements is a complex task, because the measured variable varies over time. Unlike static measurements, where uncertainty decreases with an increasing number of observations, time-dependent data introduce additional sources of variability, such as autocorrelation and instrumental drift. These factors must be accounted for when estimating the overall uncertainty of environmental measurements.

Since temperature follows a temporal trend, its uncertainty cannot be estimated simply as the standard deviation of individual measurements or by considering the number of observations, as this would not properly capture systematic variations. To obtain a more reliable estimate, a linear regression was performed for each hour of the dataset, assuming that temperature variations within each 1 h time bin followed a linear trend. The reliability of the regression was assessed using the correlation coefficient *R*, with a threshold of R>0.6 used to identify hours where a significant temperature trend was present. This value was chosen as it represents a moderately strong correlation, sufficiently robust to indicate a linear trend in the hourly temperature data [27]. If the regression was considered reliable, residuals—defined as the difference between measured values and model predictions—were computed for each hour, and their standard deviation was taken as the uncertainty associated with the hourly mean temperature in that specific hour. This method provided an uncertainty estimate that accounted for systematic variability in the data rather than treating all measurements as statistically independent. The method enabled the estimation of the uncertainty associated with each hourly averaged temperature value. Since more than 95% of the dataset yielded a fixed residual standard deviation of 0.1 °C, this value was applied uniformly across the entire dataset. Once the uncertainty for the mean temperature had been determined, the uncertainty on the temperature difference ΔT—defined as rooftop minus canyon temperature—was obtained through simple error propagation, resulting in a final value of 0.2 °C.

### 2.5. Radiation

To assess the effect of solar radiation on the temperature difference ΔT, a normalized global radiation value $R_{n}$ was also calculated. This was obtained by dividing the global radiation measured by the Davis station by the instantaneous theoretical clear-sky radiation:

(2) $$R_{n}=\frac{R_{m}}{R_{cs}}$$

where $R_{m}$ is the measured global radiation, and $R_{cs}$ is the instantaneous clear-sky radiation. This normalization ensures that values are scaled between 0 and 1, allowing for a consistent comparison of measurements, while accounting for the seasonal and diurnal variability of solar irradiance. Moreover, the normalized radiation $R_{n}$ serves as an effective indicator of sky conditions: values close to 1 are associated with clear-sky conditions, whereas lower values indicate the presence of clouds or overcast skies. This approach allows the analysis of the effect of solar radiation on ΔT under different sky conditions. The clear-sky radiation was computed using the Ineichen clear-sky model as implemented in the pvlib Python library version 0.13.0 (https://github.com/pvlib/pvlib-python/tree/v0.13.0, accessed on 8 June 2025), extracting the global horizontal irradiance (GHI) corresponding to the location and time of each observation [28].

## 3. Results

**Figure 4:**
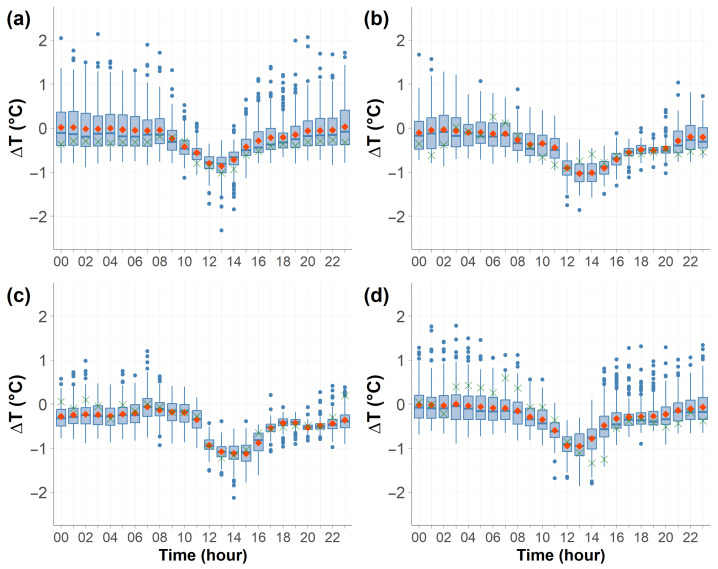
Daily trend in ΔT (defined as rooftop station minus canyon station temperature) across the four seasons, shown using boxplots: the red dot represents the mean, the blue bar indicates the median, and the green cross marks the mode. Isolated dots represent outliers, and the box extension indicates the interquartile range. Time is expressed in CET. Seasons are shown as follows: (**a**) winter, (**b**) spring, (**c**) summer, (**d**) autumn.

Figure 4 shows the daily trends in the hourly temperature differences, defined as $\Delta T=T_{\mathrm{roof}}-T_{\mathrm{canyon}}$, across the four seasons. The diurnal pattern was similar throughout the year. For most of the day, temperatures at the two stations remained closely aligned, with ΔT near zero, indicating that during these hours, the rooftop and urban canyon measurements were indistinguishable. However, during the central hours of the day (between 10:00–16:00 CET), the absolute value of the temperature differences increased, exceeding −1 °C, especially during the summer season. The maximum ΔT was observed around 13:00 CET.

During the central hours of the day, the urban canyon has difficulty dissipating the absorbed radiation due to limited ventilation, reduced sky exposure, and multiple reflections between building surfaces. This leads to heat accumulation within the canyon, resulting in a temperature increase.

In addition to the hourly trend observed during the central hours of the day, it is noticeable that during the night, particularly in winter and autumn, numerous outliers were present, all leaning toward positive values, indicating a rooftop temperature higher than the canyon.

**Figure 5:**
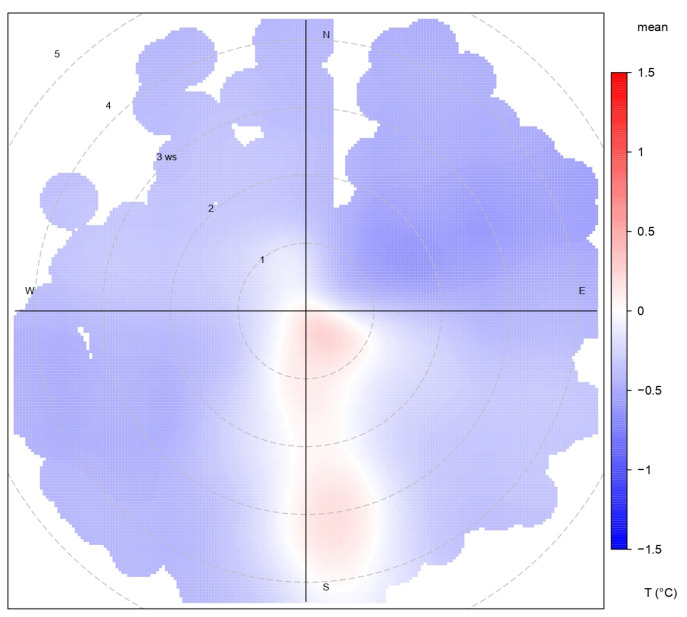
Polar plot of ΔT as a function of wind speed and direction for different ranges. The color scale represents the ΔT intensity in °C.

This pattern is further clarified by the polar plot in Figure 5, where the temperature difference during both winter and autumn is plotted as a function of wind speed and direction. The cases where the roof is warmer than the canyon (ΔT>0), represented by reddish tones, are concentrated in a specific region of the plot—namely, when wind speeds are below 4 m s^−1^ and the wind direction is from the S–SE. This suggests the occurrence of a downslope mountain breeze, generated by katabatic flow originating from the nearby Alban Hills, during clear, calm nights. Under these stable conditions, reduced vertical mixing can lead to shallow thermal stratification, with cooler air persisting in the canyon and warmer air remaining aloft. The katabatic flow transports a stratified air mass that favors the formation of a shallow thermal inversion in the lowest atmospheric layers, where temperature increases with height. This stable layering reduces turbulent exchange and explains the positive ΔT values observed under these conditions. While this interpretation is physically consistent and supported by the wind regime, it is based on indirect evidence and should be further tested with additional vertical temperature profile measurements.

In all other conditions, with higher wind speeds or different directions, the ΔT remained slightly negative, indicating that the roof was generally cooler than the canyon.

### 3.1. Influence of Solar Radiation

To assess the influence of other meteorological variables, ΔT was analyzed in relation to the solar radiation measured by the rooftop station. As previously mentioned, global radiation measurements were normalized by the instantaneous clear-sky value to obtain a dimensionless indicator of sky conditions. This normalization allowed a consistent comparison of solar forcing across different days and seasons, and made it possible to distinguish between clear-sky and cloudy-sky conditions when evaluating the radiative contribution to the temperature difference. As a result, the normalized radiation values ranged between 0 and 1, with values approaching 1 corresponding to clear-sky conditions at the time of measurement, whereas lower values indicate the presence of clouds or overcast conditions.

**Figure 6:**
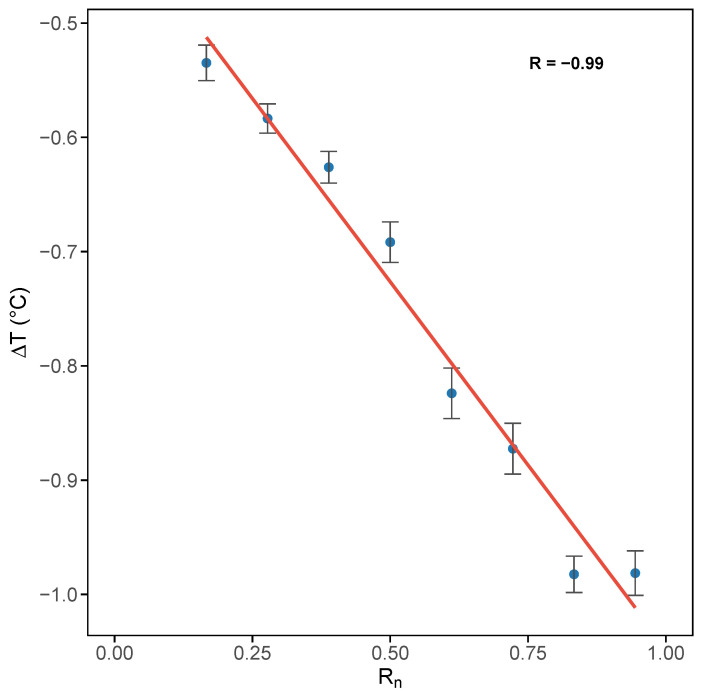
Scatter plot of ΔT (°C) versus binned normalized radiation between 13:00–15:00 CET. *R* is the correlation coefficient. Error bars indicate the standard deviation divided by the square root of the number of observations in each bin.

The anticorrelated relationship between ΔT and normalized radiation is illustrated in Figure 6, using data collected across all seasons during the time window 13:00–15:00 CET. To reduce dispersion and enhance the robustness of the analysis, each data point was derived by linearly binning normalized radiation values and excluding those with a standard deviation greater than 0.1. The same analysis was also applied to the other time windows, including morning (08:00–10:00) and evening (17:00–19:00) hours. In both cases, ΔT remained close to zero and showed no correlation with normalized radiation, indicating that solar forcing had little effect on the rooftop–canyon temperature difference during morning and evening hours.

### 3.2. Influence of Wind Speed

**Figure 7:**
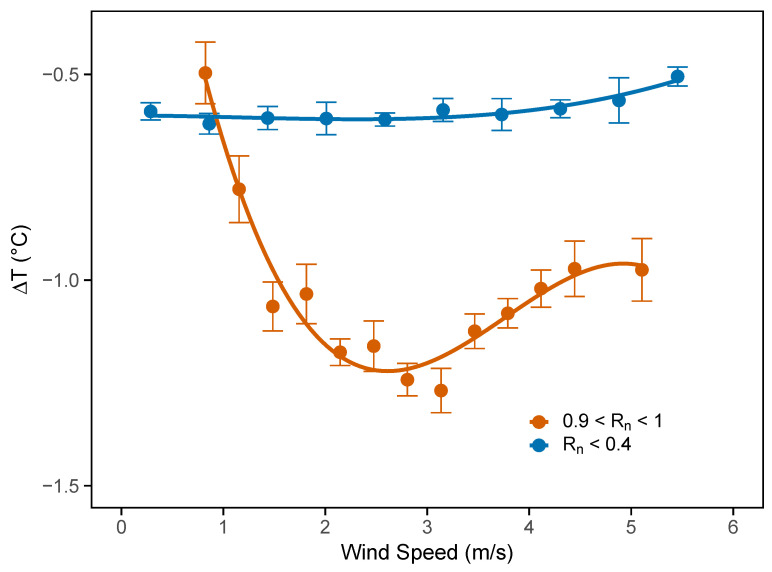
ΔT as a function of binned wind speed measured on the rooftop during the 13:00–15:00 CET time window. The orange curve represents clear-sky conditions ($0.9<R_{n}<1$), while the blue curve corresponds to cloudy-sky conditions ($R_{n}<0.4$). Error bars indicate the standard deviation divided by the square root of the number of observations in each bin.

The analysis focused on two contrasting radiative conditions, shown in Figure 7, using the same time window as in the previous plot (13:00–15:00 CET). The goal was to analyze how ΔT behaved in relation to wind speed measured on the rooftop, while keeping solar radiation fixed within two distinct regimes: normalized radiation below 0.4 (indicated in blue), representing cloudy conditions, and normalized radiation between 0.9 and 1 (indicated in orange), corresponding to clear-sky conditions. The wind speed was divided into 20 bins, and the mean ΔT was computed for each bin.

Figure 7 shows that under cloudy-sky conditions (blue curve), the wind speed did not appear to significantly affect the temperature difference between the rooftop and the canyon, which remained nearly constant around −0.5 °C. In contrast, under clear-sky conditions (orange curve), a more complex relationship between ΔT and wind speed emerged.

This behavior followed different regimes. At low wind speeds (below 1 m s^−1^), the temperature difference was small. In these conditions, even the rooftop station was affected by heat from nearby surfaces, as limited ventilation hindered air exchange around the sensor. As a result, both the rooftop and canyon were similarly influenced by their immediate surroundings.

As the wind became moderate (up to around 3 m s^−1^), the flow above the rooftop was sufficient to mix the air, reducing the effect of nearby surfaces on the rooftop sensor. Meanwhile, the canyon remained poorly ventilated: the airflow was weaker, and heat dissipation was less effective. This caused an increase in the absolute value of ΔT.

At higher wind speeds (above 5 m s^−1^), the airflow was strong enough to penetrate the canyon and promote ventilation, even at the lower sensor. In this regime, a decreasing trend in ΔT was observed as wind speed increased, indicating a positive correlation between the two variables. The same analysis was repeated for morning (08:00–10:00) and evening (17:00–19:00) hours. During these periods, the temperature difference remained close to zero across all wind speeds, suggesting that wind-driven modulation of ΔT was negligible outside the central hours of the day.

## 4. Discussion and Conclusions

This study shows that, for most of the day, measuring temperature on a rooftop or within an urban canyon yielded nearly identical results, as the temperature difference between the two stations was generally negligible. However, during the central hours of the day (12:00–16:00 CET), the situation changed, with the temperature difference between the rooftop and the canyon reaching −1 °C as the canyon became significantly warmer due to heat retention and limited ventilation. The results are consistent with those previously found by Giovannini et al. [15] and Offerle et al. [16]. This highlights how local urban geometry, solar radiation, and airflow influence temperature variations, making the choice of measurement site particularly relevant during specific periods of the day.

The analysis showed that solar radiation was the main factor driving the temperature difference between the two measurement sites. The three-dimensional geometry of the urban canyon promotes heat accumulation on internal surfaces, resulting in an increase in air temperature within the canyon. In contrast, the rooftop has a more open, two-dimensional geometry, which allows for greater heat dissipation, also facilitated by more effective ventilation due to its direct exposure to the unobstructed wind flow.

The analysis of wind speed highlighted that, under conditions of normalized radiation below 0.4 (i.e., during the time window 13:00–15:00 CET, which was associated with the highest ΔT values), corresponding to cloudy skies, the wind speed measured on the rooftop did not influence the temperature difference, which remained nearly constant at around −0.5 °C, regardless of wind intensity.

In contrast, under clear-sky conditions, with normalized radiation between 0.9 and 1, the wind speed had a significant effect on ΔT, showing a nonlinear response. The temperature difference was minimal under calm wind, reached a maximum at intermediate wind speeds, and then decreased again at higher wind intensities. This behavior confirmed the strong impact of wind on the thermal contrast between the rooftop and canyon, especially when solar radiation was high.

In summary, solar radiation was identified as the main factor influencing the temperature difference between the rooftop and canyon. Wind also played a role, but primarily under clear-sky conditions, as it helped to mix the air around the sensor located within the canyon, thereby affecting the measured temperature.

The presented results can also contribute to assessing the reliability of UHI studies, which are often performed using temperature sensors installed on rooftops rather than within the urban canyon. Since the UHI was mainly a nocturnal phenomenon—when the difference between rooftop and canyon temperature remained close to zero—the location of the sensors had a smaller impact than previously thought. More generally, the temperature measured inside an urban canyon reflects what people experience, making it especially relevant for studies on thermal comfort and urban climate impacts. However, it is strongly affected by many local factors that are difficult to quantify, such as building geometry, ventilation, shading, and surface radiation. As a result, its representativeness at the city scale is limited. In contrast, a rooftop measurement, although different from the canyon temperature during certain hours of the day, is less influenced by local microclimatic factors and provides a more reliable estimate of a city’s overall temperature.

Although this study shows that canyon temperatures can exceed rooftop values during early afternoon hours, it is also well established that appropriate canyon geometry, shading, and vegetation can help reduce temperatures at pedestrian level. From an urban design perspective, increasing the height-to-width ratio or enhancing natural ventilation has been shown to improve thermal comfort within street canyons, underscoring the relevance of detailed temperature measurements within the urban canopy [29].

Although the analysis was based on a single rooftop–canyon pair, the 21-month dataset ensured statistical robustness across seasons and weather conditions. Moreover, the observed diurnal pattern, characterized by negligible night-time differences and peak canyon warming during the day, is consistent with the results from multi-site experiments conducted in Trento (Italy) [15], supporting the broader applicability of the findings to similar urban settings. In future works, this approach could be extended to additional monitoring sites, both in denser areas of the same city and in other urban environments, to assess how different urban structures and land uses influence the temperature contrast between rooftop and canyon locations. This would help determine the most appropriate and representative placement of sensors for urban temperature monitoring across various city settings

## Data Availability

The data presented in this study are available on request from the corresponding author.

## Abbreviations

The following abbreviations are used in this manuscript:

UCL	Urban Canopy Layer
UHI	Urban Heat Island
WMO	World Meteorological Organization
